# Evaluation of the cellular impact of missense variants in low-density lipoprotein receptor-related protein 6 (LRP6) associated with cardiovascular diseases in HeLa and HEK293T cell lines

**DOI:** 10.3389/fcell.2026.1828772

**Published:** 2026-07-09

**Authors:** Anjana Raj, Huda I. Samha, Sally Badawi, Anne John, Alia Almehrezi, Mohammad A. Ghattas, Bassam R. Ali

**Affiliations:** 1 Department of Genetics and Genomics, College of Medicine and Health Sciences, United Arab Emirates University, Al-Ain, United Arab Emirates; 2 College of Pharmacy, Al Ain University, Abu Dhabi, United Arab Emirates

**Keywords:** cardiovascular diseases, LDLR-related protein 6, low-density lipoprotein receptor, metabolic syndrome, molecular dynamics

## Abstract

Low-density lipoprotein (LDL) receptor-related protein 6 (LRP6) is crucial for the canonical wingless signaling pathway and the clearance of LDL from the bloodstream. Genetic variants in the *LRP6* gene have been conclusively associated with cardiovascular diseases (CVDs) and metabolic syndrome. However, the structural, cellular, and functional implications of these variations have not been fully elucidated. In this study, we examined the subcellular localization, stability, and degradation of 10 LRP6 missense variants (K82N, R360H, Y418H, N433S, R473Q, S488Y, R611C, P1066T, P1206H, and I1264V) previously reported to be associated with various CVD conditions. We assessed the effect of these missense variants on LRP6 subcellular localization by overexpressing them in HeLa and human embryonic kidney (HEK293T) mammalian cell lines. Molecular dynamic (MD) simulation was performed on two variants to evaluate their stability. In addition, the stability of all the variants was evaluated experimentally by measuring their half-lives and comparing them to the wild-type (WT) protein, using cycloheximide chase assays and inhibitor treatments. Our findings suggest that approximately 45% of the wild-type LRP6 protein achieves its mature form within 24–48 h of overexpression, indicating its modest trafficking through the endoplasmic reticulum (ER), maturation, and transport to the plasma membrane. On the other hand, CVD-associated LRP6 variants Y418H, N433S, R473Q, and P1206H exhibited significantly lower maturation levels and, in some cases, were semi-quantitatively present in the immature form, suggesting retention within the ER and failure to pass the highly stringent ER quality control systems. The *in silico* stability assessment revealed that all 10 LRP6 missense variants are predicted to have a negative impact on protein stability. Interestingly, MD simulation elaborated that one fully ER-retained variant, P1066T, has altered structural interactions of the protein, affecting its folding. ER retention of some CVD-associated LRP6 variants could contribute to diseases via the reduction in LRP6 plasma membrane localization and consequently loss or reduction of LRP6 function, potentially leading to dysregulated signaling efficiency. This study contributes to improving our understanding of the cellular behavior of several LRP6 missense variants causing CVD conditions and has potential applications in diagnosis and the development of new therapies for their associated conditions.

## 1 Introduction

The global increase in cardiovascular diseases (CVDs) is driven by various risk factors, including behavioral, physiological, demographic, and genetic influences (Eaton, 2005). These factors often combine to form metabolic syndrome (MetS), a cluster of conditions that include hyperglycemia/insulin resistance, abdominal obesity, atherogenic dyslipidemia, and endothelial dysfunction (Huang, 2009). This syndrome eventually may lead to the development of atherosclerosis and progression of CVDs (Olver et al., 2026). Extensive genetic screening and genome-wide association studies have been exploited to identify monogenic and polygenic traits correlated with more than two metabolic syndrome symptoms and the early-onset CVDs (Cao et al., 2018; Khera et al., 2019; Abou Ziki and Mani, 2016). Genetic variations in the low-density lipoprotein receptor (LDLR)-related protein 6 (LRP6), a member of the LDLR protein family, have been shown to be conclusively associated with cardiometabolic diseases (Xu et al., 2014; Singh et al., 2013; Mani et al., 2007).

**FIGURE 1:**
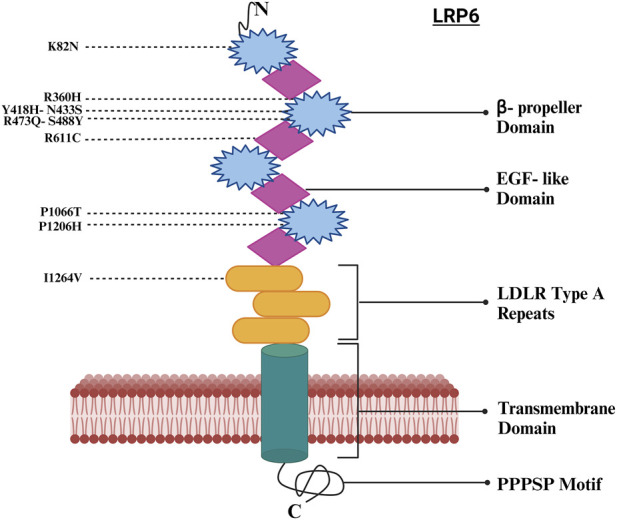
*A diagram of LRP6 protein structure and the CVD-associated mutation sites on the LRP6 protein.* A schematic representation of the domain structures of LRP6 protein. The extracellular domains include LDLR type A repeats, EGF homology region, and a YWTD type-β propeller domain; however, each has a distinct signaling sequence in the cytoplasmic domain. EGF, epidermal growth factor; YWTD-Tyr-Trp, NPxY motif- Thr-Asp.Asn-Pro-x (any amino acid)-Tyr, PPPSP motif-Pro-Pro-Pro-Ser-Pro EGF-Epidermal Growth Factor, NPxY-Asparagine-Proline-x (any amino acid)-Tyrosine motif. All of the 10 studied variants are located on the extracellular region of the protein. Figure generated using biorender.com.

*LRP6* is a highly conserved gene across different species, located on chromosome 12p13.2 in humans. It spans 150 kb and consists of 23 exons (Brown et al., 1998). The *LRP6* gene encodes for a type 1 transmembrane protein composed of 1,613 amino acids, which is highly expressed in the heart, pancreas, placenta, ovary, lung, brain, kidney, and other tissues (Brown et al., 1998). Homozygous deletion of the *LRP6* gene in transgenic mice results in perinatal lethality, suggesting its crucial role in viability (Pinson et al., 2000). Structurally, the LRP6 protein is composed of six main types of domains, namely, LDLR type A repeats, EGF homology regions, β-propeller domains, an O-linked sugar domain, a transmembrane domain, and a C-terminal cytoplasmic domain (Figure 1).

In adults, LRP6 plays an essential role in LDLR internalization and various signaling pathways, many of which are related to the development of CVDs (Kang, 2020). However, the primary role of LRP6 is to function as a co-receptor in the canonical Wnt/β-catenin signaling pathway, where cytosolic β-catenin translocates to the nucleus and recruits co-transcription factors that initiate the transcription of downstream targets (Labbé and Thorin, 2019). Other functions of LRP6 include the clearance of LDL from the plasma and protecting from the development of hyperlipidemia and atherogenic lesions. LRP6 endocytosis of LDL occurs either by independent binding and uptake of LDL or through the formation of a complex with LDLR (Liu et al., 2008; Ye et al., 2012; Go and Mani, 2012). LRP6 also regulates fatty acid utilization in cardiomyocytes by preventing the phosphorylation of dynamin-related protein 1 (DRP1), a kinase that regulates the autophagy of mitochondria in response to lipid accumulation and mitochondrial dysfunction (Ikeda et al., 2015; Ren et al., 2010). This regulatory function is impaired in patients with end-stage dilated cardiomyopathy, due to significant downregulation of LRP6 expression (Wang et al., 2020). In mice, cardiac-specific deletion of *LRP6* results in impaired myocardial autophagy, lipid accumulation, and, consequently, acute heart failure and lethality (Ikeda et al., 2015; Wang et al., 2020). Genetic screening studies identified 10 *LRP6* variants associated with coronary artery disease (CAD), hypercholesterolemia, and several metabolic syndrome (MetS) symptoms (Table 1). These variants are predominantly loss-of-function mutations. For example, the R611C variant has been shown to reduce LDL uptake compared to wild-type LRP6 (Liu et al., 2008) and impair Wnt signaling, which activates the IGF1–Sp1–mTOR–SREBP1/2 pathway, leading to elevated hepatic lipogenesis, along with increased triglyceride and cholesterol synthesis (Go et al., 2014). Other variants, such as K82N, Y418H, S488Y, P1066T, P1206H, and I1264V, have also been reported to impair Wnt signaling, affecting the cellular proliferation and migration of endothelial cells (Xu et al., 2014).

**TABLE 1:** List of LRP6 CVD-associated mutations and the exhibited phenotypes.

Mutation	Genotype	Associated phenotype	References
R611C	Autosomal dominant	Early-onset CAD, myocardial infarction, hyperlipidemia, glucose intolerance, hypertension, osteoporosis, impaired LDL clearance, increased hepatic lipogenesis, and also increased VSMC proliferation and plasticity	Mani et al. (2007), Keramati et al. (2011) Srivastava et al. (2015), Liu et al., (2008), and Ye et al. (2012)
K82N	-	Hyperlipidemia, early onset CADs, and impaired endothelial function	Xu et al. (2014)
S488T			
P1066T			
P1206H			
I1264V			
R360H	Autosomal dominant	CAD, hyperlipidemia, hypertriglyceridemia, hypertension, and glucose intolerance	Singh et al. (2013)
N433S	Autosomal dominant	CAD, hypertension, elevated LDL and triglycerides, and glucose intolerance	Singh et al. (2013)
R473Q	Autosomal dominant	Early-onset CAD, hyperlipidemia, hypertriglyceridemia, hypertension, diabetes, and osteoporosis	Singh et al. (2013)
Y418H	-	CAD, hypertension, and diabetes	Guo et al. (2016)

Although some functional analyses were conducted to evaluate the effect of some of these variations on LRP6, their impact on protein folding, trafficking, and cellular localization remains unclear. Genetic variants affecting protein folding and post-transcription modification often lead to the retention and accumulation of the misfolded proteins in the endoplasmic reticulum (ER). This triggers ER stress and unfolded protein response (UPR), promoting the degradation of these glycoproteins (Rutkowski and Kaufman, 2004). Pathogenic variants in the LDLR protein family have been previously reported to cause protein misfolding and ER retention of LDLR (Sørensen et al., 2006; Galicia-Garcia et al., 2020; Jawabri et al., 2024; Varghese et al., 2023; Barbosa et al., 2023) and very-low-density lipoprotein receptor (VLDLR) (Azmanov et al., 2013). The retention of these proteins induced ER stress and involved different ER-associated degradation (ERAD) components, such as XBP‐1 (Kizhakkedath et al., 2019) and the ER degradation adaptor protein SEL1L (Kizhakkedath et al., 2018; Badawi et al., 2023).

Therefore, understanding the molecular and cellular mechanisms underlying atherosclerosis and MetS progression is crucial for the development of new therapeutic drugs and prevention strategies (Oommen et al., 2020). The genetic or pharmacological inhibition of ERAD might rescue the transport defective mutants and restore their ER–Golgi transport, resulting in the normal expression of LRP6. Accordingly, this study employs a combination of various *in vitro* experiments and *in silico* predictions using molecular dynamic simulations to investigate the effect of 10 LRP6 genetic variants on the subcellular localization, maturation, glycosylation profile, degradation, and stability of LRP6.

## 2 Materials and methods

### 2.1 Antibodies

The antibodies with their dilutions and sources were as follows: antibodies for immunofluorescence: mouse monoclonal anti-FLAG [1:1000; F1804; Sigma], rabbit polyclonal anti-calnexin [CNX, 1:50; sc-11397; Santa Cruz Biotechnology, Dallas, TX, USA], Alexa Fluor 555-anti-mouse IgG [1: 200; 4409S; Cell Signaling Technologies (CST; Danvers, MA, USA)], and Alexa Fluor 488-anti rabbit IgG [1:200, 4412S; CST; Danvers, MA, USA]. For Western blotting, the primary antibodies and their dilutions were as follows: rabbit polyclonal anti-DYKDDDDK-tag [1:1000; S2368; CST; Danvers, MA, USA]; as a loading control for Western blot, the antibody used was rabbit polyclonal anti-α-tubulin [1: 1000; S2144; CST; Danvers, MA, USA)]. The secondary antibodies and their dilutions were as follows: anti-rabbit IgG polyclonal goat antibody [1: 40,000; A0545; Sigma].

### 2.2 Generation of *E. coli* competent cells

*Escherichia coli* competent cells were generated to be used in bacterial transformation with a foreign DNA or plasmid into the host cell for plasmid amplification. JM109 E. *coli* competent cells were generated following the protocol proposed by Chang et al. (2017).

### 2.3 Primer’s design and site-directed mutagenesis for the generation of LRP6 missense variants

The human LRP6 ORF clone inserted into the pCMV6-Entry Myc-DDK-tagged plasmid was obtained from OriGene Technologies, Inc. (Rockville, MD, USA), CAT#: RC218918. To create a specific missense mutation in the double-stranded plasmid, mutagenic primers for the selected mutations were designed using the Primer X online tool (https://www.bioinformatics.org/primerx/) (Supplementary Table S1). All the mutations described in this study are with reference to the coding LRP6 sequence represented by GenBank accession number GRCh38/hg38—NM_002336.2 and the protein sequence represented by NP_002327.2. Site-directed mutagenesis was performed using PfuUltra HF polymerase (Stratagene, La Jolla, CA, USA). For elimination of the methylated parental DNA, PCR samples were digested with the Dpn1 enzyme (Invitrogen) at 37 °C for 4 h.

To verify the mutagenesis of the generated LRP6 plasmids, DNA sequencing was performed using the Sanger dideoxy method by fluorescent automated sequencing on the ABI 3130xl Genetic Analyzer (Applied Biosystems, Waltham, MA, USA). The primers used for sequencing are listed in Supplementary Table S2. The sequence of each mutant plasmid was aligned with the wild-type plasmid using Clustal Omega software (https://www.ebi.ac.uk/Tools/msa/clustalo/).

### 2.4 Cell culture and transfection

HeLa and human embryonic kidney (HEK293T) cells (HEK-293T; ATCC, Manassas, VA, USA) were cultured in Dulbecco’s modified Eagle’s Medium (DMEM; Invitrogen, Carlsbad, CA, USA) supplied with 10% fetal bovine serum (FBS; Invitrogen) and 100 U. ${\text{ml}}^{-1}$ penicillin/streptomycin, at 37 °C with 5% CO2. For immunostaining and Western blot analysis, HeLa cells and HEK293T at 70% confluency were split by aspirating DMEM, followed by washing the DMEM residue with phosphate-buffered saline (PBS; Gibco™). The cells were detached using trypsin (Gibco™) for 5 min at 37 °C in a sterile cell culture incubator. An aliquot of 5 mL DMEM was added to stop the action of trypsin, and cells were passaged to a new flask containing fresh DMEM.

### 2.5 Immunocytochemistry and confocal microscopy

To study the cellular localization of mutant LRP6, HeLa cells were grown over sterilized coverslips in a 24-well tissue culture plate, and then, transient transfection was performed. For each well, a mixture of 25 μL of Opti-MEM media (Thermo Fisher), 3 μL of FuGene HD transfection reagent (Promega, Madison, WI, USA), and 1 μg of Myc-DDK-tagged wild-type or mutant LRP6 constructs was prepared. The GFP-HRas plasmid was used as a plasma membrane marker and co-transfected with the LRP6 wild-type or mutant plasmids (Ali et al., 2010). The transfected HeLa cells were incubated at 37 °C for 24 h before proceeding to the immunostaining and imaging.

Twenty-four hours after transfection, the DMEM media was aspirated and then washed with PBS. The cells were fixed with −20 °C methanol (Fisher Chemical) for 5 min. The fixed cells were washed again with PBS, and then, blocking was performed using 1% bovine serum albumin (BSA) (Pierce™) for 1 hour at room temperature. After blocking, HeLa cells were incubated with primary antibodies for 1 h at room temperature in mouse monoclonal anti-FLAG-tag (Sigma) and rabbit polyclonal anti-calnexin (Santa Cruz Biotechnology). Afterward, the cells were washed with PBS and incubated with Alexa Fluor 555-anti-mouse IgG (CST) and Alexa Fluor 488-anti rabbit IgG (CST) secondary antibodies for 45 min at room temperature. Finally, the cells were mounted on cover slides using an immunofluor mounting medium (ICN Biomedicals).

Confocal microscopy and imaging were performed using the Nikon Eclipse system (Nikon Instruments Inc) containing FITC and TRITC filters. Images were captured using a 100× oil immersion objective lens. All images presented are single sections in the z-plane. The scale bar is 20 μm. This process was repeated in three independent experiments to confirm reproducibility.

### 2.6 Western blot analysis for N-glycosylation profile analysis

HEK293T cells were seeded in six-well tissue culture plates and then cultured for 24 h. Transfection was performed using 3 μL FuGene HD (Promega) and 1 μg of wild-type or mutant LRP6 plasmids. After 48 h of transfection, HEK293T cells were lysed in a mixture of RIPA buffer (ThermoScientific™) and protease phosphatase inhibitor cocktail (100x; ThermoScientific™) at 4 °C. Protein estimation was performed using the bicinchoninic acid protein assay (BCA kit, Pierce), following the manufacturer’s instructions.

For Western blotting, 20 μg of the protein from each sample was prepared with 5x Laemmli buffer and then loaded and run in a 6% acrylamide gel (ThermoFisher), followed by blotting onto the PVDF membrane (Immobilon®-P). The membrane was incubated with the respective primary antibodies: monoclonal anti-DYKDDDDK-tag (CST) and rabbit monoclonal anti-a-tubulin (CST), followed by incubation with the respective secondary antibody: anti-rabbit IgG goat antibody (Sigma). Detection and development were performed using the Enhanced Chemiluminescence plus kit (ECL plus kit; ThermoFisher™) and the Typhoon FLA 9500 Imager (GE Healthcare Biosciences). Images were edited on ImageJ software. Statistical significance was assessed for four independent experiments using one-way ANOVA followed by Dunnett’s multiple-comparisons test using GraphPad prism software (San Diego, CA, USA).

For performing the endoglycosidase H sensitivity assay, the protein lysates of HEK293T cells were prepared using the Endoglycosidase H Assay Kit [A0810-sigma], following the manufacturer’s instructions. Each sample was then divided into two equal aliquots, which were incubated for 3 h at 37 °C in the presence or absence of 10 U of endoglycosidase H (Endo H) [A0810; sigma]. The digested samples were then resolved on 6% SDS/PAGE gels and analyzed using Western blotting as described above.

### 2.7 Molecular dynamic simulation

The crystal structures of the E1E2 and E3E4 domains of LRP6 were obtained from the Protein Data Bank [PDB ID: 3S94 (Cheng et al., 2011) and 4A0P (Chen et al., 2011), respectively]. The LRP6 structures were inspected and prepared using MOE software. Protein preparations included adding missing atoms and residues; assigning protonation states for ionizable side chains; and assigning partial charges for all protein atoms (MOE, 2020).

The previously prepared crystal structures of LRP6 were further refined via conducting MD simulation for 100 ns. This included creating their topology and coordination files via XLeap in AmberTools and then conducting a two-step minimization process (1,000 steps each) using the pmemd program in Amber18 (Case et al., 2018). Only the first minimization step had restraint forces of 500 kcal/mol applied on the protein atoms. The system was then heated from 0 to 300 K under NVT conditions for 20 ps, using Langevin thermostat and restrain forces on protein atoms of 500 kcal/mol. Afterward, the density of the system was equilibrated for 100 ps, where the solute atoms were restrained with 2 kcal/mol forces.

A total of 100 ns MD simulations were conducted via PMEMD in AMBER18, storing the generated coordinates every 2 ps. The production stage was carried out under NPT conditions, with an average pressure of 1 atm and a relaxation time of 2 ps. The system average temperature was set at 300 K and was managed using a Langevin thermostat (using a collision frequency of 1.0 ps^-1^). All explicit solvent calculations were carried out via Particle Mesh Ewald (Darden et al., 1993), with a cutoff of 10 Å for long-range electrostatics. The SHAKE algorithm was selected to constrain hydrogen atoms’ covalent bonds with a time step of 2 fs. Clustering was performed using the CCPTRAJ module in AmberTools (Shao et al., 2007). Frames were previously stripped out of ions and solvent molecules. The epsilon value was set to 3.

### 2.8 Stability study

The Residue Scan module in MOE (Molecular Operating Environment (MOE), 2020) was then used to create each mutant form and assess its stability compared to that of the wild-type form via calculating the ΔStability parameter. Each mutant form was assessed for pre-mutation and post-mutation interactions through the ligand interaction module in MOE. The above MD simulation protocol was applied for two interesting variants (i.e., R611C and P1066T); then, post-MD analysis was carried out to monitor the protein backbone and detect any changes in its secondary structure.

### 2.9 Cycloheximide (CHX) assay for LRP6 half-life study and inhibitor treatments

HEK293T cells, once they reached 70%–80% confluence, were transiently transfected with WT or LRP6 plasmid constructs. After 24 h, the cells were treated with cycloheximide (CHX, 100 μg/mL) for different time points (0, 2, 3, 4, 6, 7, and 12 h) to stop new protein translation. DMSO-treated cells were taken as a control. Cells were lysed in RIPA buffer, and the LRP6 protein was assessed using Western blot and normalized to alpha-tubulin expression. Densitometry analysis was performed using ImageJ software. Three independent experiments were conducted, and data were analyzed using GraphPad Prism.

For treatment with proteasomal or lysosomal inhibitors, HEK293T cells were transiently transfected with WT or LRP6 variants. After 24 h, transfected with WT/ variants, cells were serum-starved for 4–6 h. The cells were then treated with DMEM media containing the indicated proteasomal inhibitors (MG132 at 10 µM and epoxomycin at 100 nM), ERAD inhibitor (kifunensine at 50 nM), and lysosomal inhibitor (bafilomycin at 200 nM) for 16 h. Experiments were conducted in three independent replicates. Data analysis was performed using GraphPad Prism, and statistical significance for each treatment relative to DMSO was assessed using two-way ANOVA.

## 3 Results

### 3.1 Several disease-associated missense LRP6 variants exhibited aberrant subcellular localization

**FIGURE 2:**
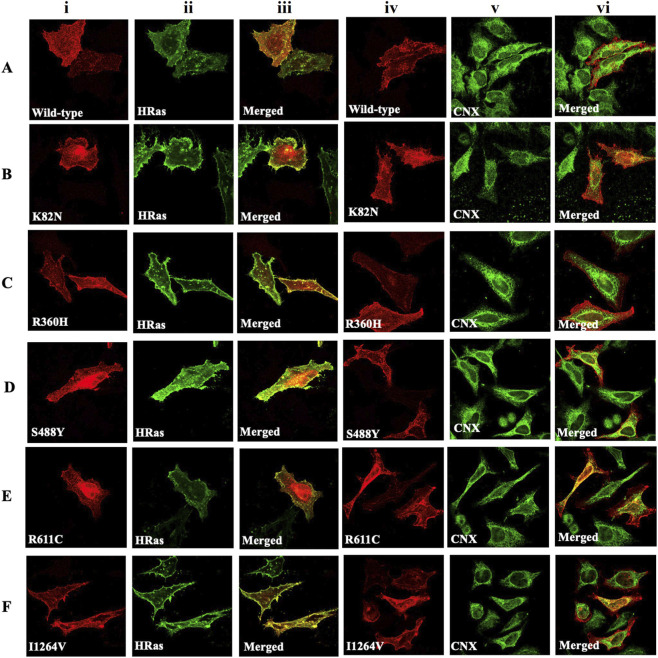
Confocal images of WT and variants of LRP6 that are expressed completely or partially along the plasma membrane. HeLa cells co-transfected with WT **(A)**/variants of LRP6 **(B–F)** and GFP-tagged HRas plasmid and immuno-stained with LRP6/Calnexin antibodies. GFP-tagged HRas was used as a plasma membrane marker, and calnexin (CNX) as the ER marker. Wild-type LRP6 co-expresses with HRas on the plasma membrane. Similar to the wild type, the LRP6 variants K82N, R360H, and S488Y co-localize with HRas on the plasma membrane, but not with the calnexin in the ER. The mutants R611C and I1264V localize both in the ER, along with calnexin, and on the plasma membrane, with GFP-tagged HRas. The panels representing the green, red, or merged channels are as follows: (i), (iv) expression of FLAG-tagged WT-/variants of LRP6; (ii) GFP-tagged HRas along the plasma membrane; (iii) merged images of FLAG- and GFP-tagged proteins; (i), (ii) illustrating the co-localization of LRP6 and HRas; (v) calnexin; (vi) merged images of FLAG-tagged and calnexin expression. Images were captured using the ×100 oil immersion objective of a Nikon confocal microscope equipped with TRITC and FITC filters. Image contrast was adjusted using ImageJ software.

The selected 10 LRP6 variants displayed in Figure 1 (K82N, R360H, Y418H, N433S, R473Q, S488Y, R611C, P1066T, P1206H, and I1264V) associated with human diseases have been generated by site-directed mutagenesis, as described in the *Methods* section and confirmed by Sanger DNA sequencing. To inspect their impact on the subcellular trafficking and localization of the mutated LRP6 protein, the generated plasmids were transfected into HeLa cells, and the expressed proteins were evaluated using immunofluorescence confocal microscopy. The cells were transfected with the wild-type or mutant LRP6 constructs individually or co-transfected with a GFP–HRas plasmid, which was used as a plasma membrane marker. As a transmembrane protein, wild-type LRP6 exhibited the predicted co-localization with GFP–HRas on the plasma membrane (Figure 2Ai-iii), with limited co-localization with the ER marker, calnexin (Figure 2Aiv-vi).

Among the 10 evaluated variants, K82N, R360H, and S488Y showed similar subcellular localization to that of the wild-type protein. These three mutant proteins seemed to transport and localize effectively to the plasma membrane in similar levels to WT, as evidenced by the degree of their co-localization with HRas (Figures 2Bi–iii, Ci–iii, Di–iii, Ei–iii, Fi–iii). In addition, these LRP6 variants did not co-localize significantly with calnexin (Figures 2Biv–vi, Civ–vi, Div–vi). This indicates that these variants (K82N, R360H, and S488Y) did not affect the cellular trafficking of the protein nor caused ER retention. In contrast, two variants R611C and I1264V showed significant localization in both the ER network and the plasma membrane (Figures 2Eiv–vi, Fiv–vi), suggesting their partial retention in the ER or delayed subcellular trafficking and maturation.

**FIGURE 3:**
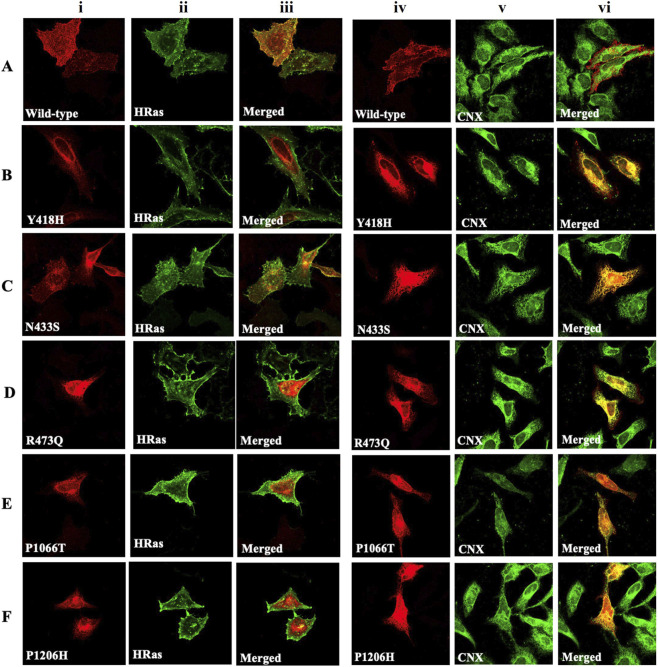
Confocal images of WT LRP6 and variants that are retained in the ER partially along the plasma membrane. HeLa cells co-transfected with WT **(A)**/variants of LRP6 **(B–F)** and GFP-tagged HRas plasmid and immuno-stained with LRP6/calnexin antibodies. GFP-tagged HRas was used as a plasma membrane marker, and calnexin (CNX) as the ER marker. Wild-type LRP6 co-expresses with HRas on the plasma membrane. Five LRP6 variants Y418H, N433S, R473Q, P1066T, and P1206H were not expressed along the plasma membrane but co-localized with calnexin in the ER. Co-transfection and immunostaining of wild-type LRP6 or the mentioned mutant plasmids with HRas into HeLa cells showed co-localization of HRas and these mutant proteins, similar to the wild type. The panels representing the green, red, or merged channels are as follows: (i) fluorescent staining and expression of FLAG-tagged LRP6; (i), (iv) expression of FLAG-tagged WT/variants of LRP6; (ii) GFP-tagged HRas along the plasma membrane; (iii) merged images of FLAG- and GFP-tagged proteins; (i), (ii) illustrating the co-localization of LRP6 and HRas; (v) calnexin; (vi) merged images of FLAG-tagged and calnexin expression. Images were captured using the ×100 oil immersion objective of a Nikon confocal microscope equipped with TRITC and FITC filters. Image contrast was adjusted using ImageJ software.

Conversely, the other five variants (Y418H, N433S, R473Q, P1066T, and P1206H) showed contrasting localization patterns to that of the wild-type protein, with no significant co-localization with GFP–HRas at the plasma membrane (Figure 3Bi-iii,Ci-iii,Di-iii,Ei-iii,Fi-iii) compared to the LRP6 WT (Figure 3Ai-iii). Crucially, these variants co-localized with calnexin in the ER (Figure 3Biv-vi,Civ-vi,Div-vi,Eiv-vi,Fiv-vi). This localization pattern suggests that these variants might have affected the folding and subcellular trafficking of these mutant proteins and led to their retention in the ER.

### 3.2 The ER-retained LRP6 variants exhibited altered glycosylation maturation profiles suggesting lower maturation and plasma membrane localization

**FIGURE 4:**
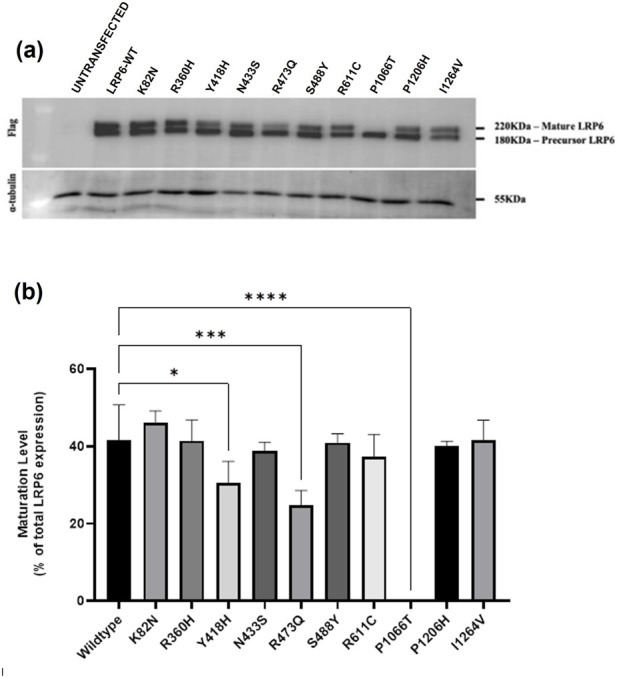
*Western blotting analysis of the maturation profile of mutant LRP6.* **(a)** Western blot for the overexpressed LRP6 protein shows two bands for the wild type, while some mutations showed lower maturation rates of the larger band than the wild type. **(b)** The graph represents the percentage of the mature protein from the total overexpressed protein. Error bars represent the ±SEM of four independent experiments; (*) P ≤ 0.02; (***) P ≤ 0.0002; (****) P ≤ 0.0001 using one-way ANOVA followed by Dunnett’s multiple-comparisons test.

To further investigate and validate the immunofluorescence results of the studied LRP6 missense variants, the flag-tagged WT and mutant LRP6 were exogenously expressed in HEK293T cells. LRP6 undergoes N-linked glycosylation, where it possesses 10 N-glycosylation sites. Immunoblotting analysis by SDS-PAGE showed that the overexpressed WT LRP6 consisted of two distinct protein bands. A lower-molecular-weight band with a size of ∼180 KDa, which presumably represents the immature unglycosylated LRP6 that is still within the ER, and a larger-molecular-weight band with a size of ∼210 kDa, representing the fully glycosylated mature protein that has been exported out of the ER, matured in the Golgi and transported to the plasma membrane (Figure 4a). In parallel and in a similar fashion, protein extracts of HEK293T cells overexpressing the studied LRP6 variants were subjected to Western blotting to investigate their maturation efficiency (Figures 4a,b).

The untransfected cell lysates represent the negative control, which was prepared to ensure that no nonspecific binding occurred. The steady state of expression after 48 h showed that approximately 45% of WT LRP6 is in the mature form, whereas 55% is in the immature form. The mutant proteins Y418H, N433S, R473Q, and P1206H, which appeared in the immunofluorescence imaging to be localized in the ER, were observed to have a much lower intensity of the mature band than the wild type. The other variants K82N, R360H, S488T, R611C, and I264V showed both lower and upper bands at variable ratios compared to the WT. In contrast, the variant P1066T showed only the lower-molecular-weight immature band at ∼180 KDa, suggesting its quantitative retention in the ER, which is presumably mediated by ERAD components (Figure 4b; Supplementary Image 1).

**FIGURE 5:**
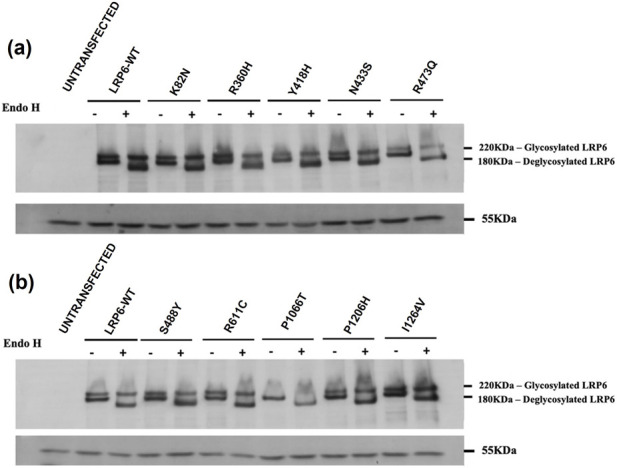
**(a,b)** *Endo H analysis of LRP6 variant glycosylation*. HEK293T cells were transfected with LRP6 wild-type or mutant plasmids, followed by treatment with endoglycosidase H (+) or left untreated (−) for 3 h at 37 °C and subsequently analyzed using Western blotting. The lower band representing the LRP6 in the ER was sensitive to Endo H treatment for both wild-type and mutant proteins.

To study the glycosylation profiles of LRP6 WT and variants, the protein lysates of HEK293T cells transfected with LRP6 WT and the different variants were subjected to endoglycosidase H (Endo H) digestion and analyzed using Western blotting. The Endo H enzyme digests the N-linked oligosaccharides of the high-mannose and hybrid (pre-Golgi) forms exclusively but is unable to digest the complex mature carbohydrate structures that form when glycoproteins are exported through the Golgi. Treatment with Endo H resulted in the digestion of the N-glycans of immature lower-molecular-weight bands as they migrated lower within the SDS-PAGE gel. On the other hand, the mature band with completed N-glycosylation was resistant to Endo H digestion, and therefore, its migration on SDS-PAGE gels did not change due to the treatment. As shown in Figure 5, the variants with lower maturation rates (Y418H, N433S, R473Q, and P1206H) were affected by the Endo H treatment, consistent with their immuonofluorescence localization results that exhibited ER retention (Supplementary Image 2).

### 3.3 The 10 selected disease-associated LRP6 variants affected the stability and, in some cases, the structural interactions of the protein

To gain further insights on the possible impact of those variations on LRP6, computational assessment was carried out to test and visualize the effect of the studied mutations on the surrounding interaction network and thus their potential impact on the protein structure and folding. First, the crystal structures of LRP6 were examined for any broken loops or missed residues, which were then automatically corrected via the protein preparation module. Next, the corrected structures were further refined through 100-ns molecular dynamics simulations. The trajectories were then clustered, and the central conformation of the top cluster ensembles was extracted to serve as the wild-type structure in subsequent stability study of the LRP6 mutations.

Mutations were introduced individually, and the Δstability energies were calculated and are listed in Table 2. Interestingly, all mutations exhibited a positive Δstability energy, indicating a negative impact on overall protein stability. To explore this further, each mutated residue was visualized to monitor changes in interactions that might be responsible for the structural instability. Table 2 illustrates those interactions lost or newly formed due to the respective mutation.

**TABLE 2:** The Δstability energies of nine mutant forms of LRP6.

Domain	Mutation	Δ stability (Kcal/mol)	Side-chain interaction	Net change
BP1E1	Wild-type	_	Ionic interaction with Glu 78	New bond formed
	K82N	0.68	H-bond with Thr83	
BP2E2	Wild-type	_	Ionic interaction with Asp358	One bond lost
	R360H	1.7	H-bond with Asp358	
BP2E2	Wild-type	_	H-bond with Leu476	One bond lost
	Y418H	2.45	No side-chain interaction	
BP2E2	Wild-type	_	H-bond with Arg386	One bond lost
	N433S	0.81	No interaction	
BP2E2	Wild-type	_	Ionic interaction with Asp520	Two bonds lost
	R473Q	1.9	No interaction	
BP2E2	Wild-type	_	H-bond with Lys513	No change
	S488Y	1.2	H-bond with Lys513	
BP2E2	Wild-type	_	Ionic interaction with Asp477	Two bonds missed
	R611C	1.32	No interaction	
BP4E4	Wild-type	_	No interaction	New bonds formed
	P1066T	0.54	Pi interaction with His1205; H-bond with Asp1022	
BP4E4	Wild-type	_	No interaction	New bond formed
	P1206H	1.09	H-bond with Gly1212	

**FIGURE 6:**
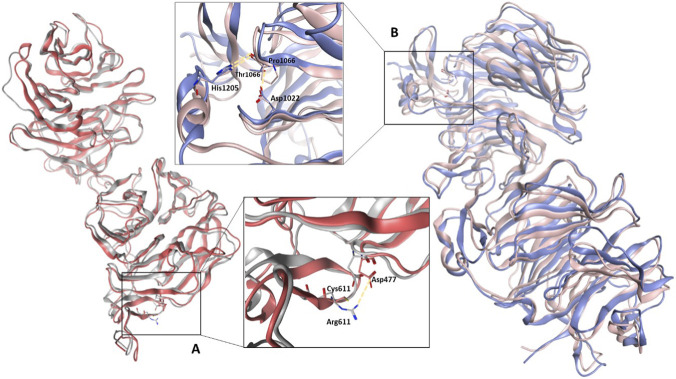
*MD simulation illustrates the effect of two LRP6 variations, R611C and P1066T, affecting different interactions.* **(A)** The MD simulation-refined structures of BP1E1–BP2E2 domain’s wild-type (silver) and mutant form (red) aligned with each other, along with a zoom-in picture for the lost hydrogen bond that was lost with Asp477 upon the R611C mutation; **(B)** The MD simulation-refined structures of BP3E3–BP4E4 domain’s wild-type (pink) and mutant form (blue) aligned with each other, along with a zoom-in picture for the newly formed hydrogen bond with Asp1022 that took place upon the P1066T mutation.

**FIGURE 7:**
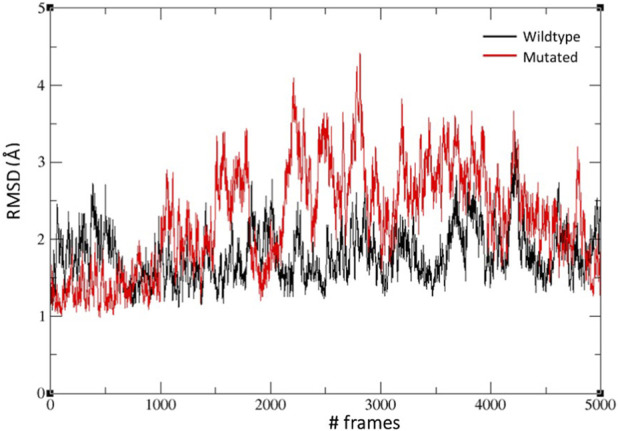
*The root mean square deviation (RMSD) plot of the wild-type and the P1066T mutant form of the LRP6 BP3E3*–*BP4E4 domain.* The RMSD of backbone atoms over simulation time is shown, with the wild type represented in black, whereas the mutant in red.

Two particularly interesting mutant forms, R611C and P1066T, were studied further through 100-ns MD simulation. Although R611C resulted in the loss of an interaction with Asp477, P1066T involved the mutated residue forming a new interaction with Asp1102 (Figure 6). In terms of overall alignment with the wild type, R611C appears to align well, while P1066T shows a clearer deviation from the wild type. To investigate this further, the RMSD plot was generated for the latter mutation. As shown in Figure 7, P1066T exhibits a relatively high RMSD average of 2.79 Å, fluctuating between 1.0 and 4.4 Å, which explains the observed imperfect alignment shown in Figure 6.

### 3.4 The overexpressed ER-retained LRP6 mutants exhibited slightly lower half-lives than the WT protein

**FIGURE 8:**
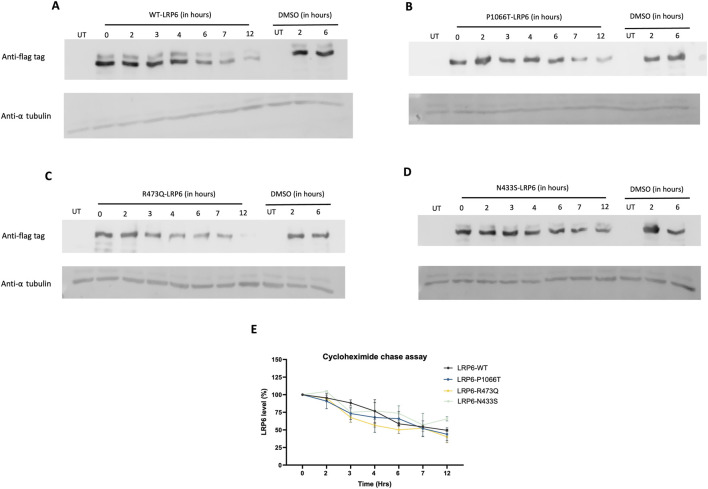
Cycloheximide (CHX) chase assay for transient transfected HEK293T LRP6 WT/mutants. **(A–D)** HEK293T cells were transiently transfected with LRP6 WT, p. P1066T, p. R473Q and p. N433S, treated with cycloheximide (100 μg/mL) for the time points 0, 2, 3, 4, 6, 7, and 12 h. DMSO treated for 2 and 6 h. **(E)** Line graph for LRP6 the cycloheximide chase assay represents mean densities of WT LRP6 and mutants relative to untreated at 0 h, normalized with loading control (alpha tubulin). Error bars represent SEM from three independent experiments.

To assess the half-lives and turnover of both WT and variant LRP6, we expressed the proteins in HEK293T cells for 24 h, then added the protein synthesis inhibitor cycloheximide at 100 μg/mL, and quantified the remaining LRP6 by Western blotting at several subsequent time point intervals for up to 12 h. As shown in Figure 8, overexpressed WT LRP6 has an approximate half-life of 12 h, whereas the mutants P1066T, N433S, and R473Q showed slightly lower half-lives, indicating that these variants might affect the protein’s stability. The densitometric analysis of the bands showed a time-dependent decrease in protein levels (Supplementary Image 3).

#### 3.4.1 Degradation pathway analysis by different inhibitors

To investigate the possible degradation routes of the two ER-retained variants and compare them to WT, LRP6 WT and mutants were transiently transfected into HEK293 cells for 24 h. The cells were then treated with bafilomycin (200 nM) (lysosomal inhibitor), kifunensine (50 nM) (ERAD inhibitors), MG132 (10 μM), and epoxomycin (100 nM) (proteasomal inhibitors) and cultured for 16 h. After 16 h, the cells were lysed in RIPA buffer, and cell lysates were analyzed by Western blotting analysis. To depict the accumulation level of LRP6 compared to DMSO-treated cells, densitometric analysis was carried out. Immunoblotting analysis has shown an accumulation of both LRP6 WT and the variants P1066T, R473Q, and N433S after treatment with MG132, a proteasomal inhibitor.

**FIGURE 9:**
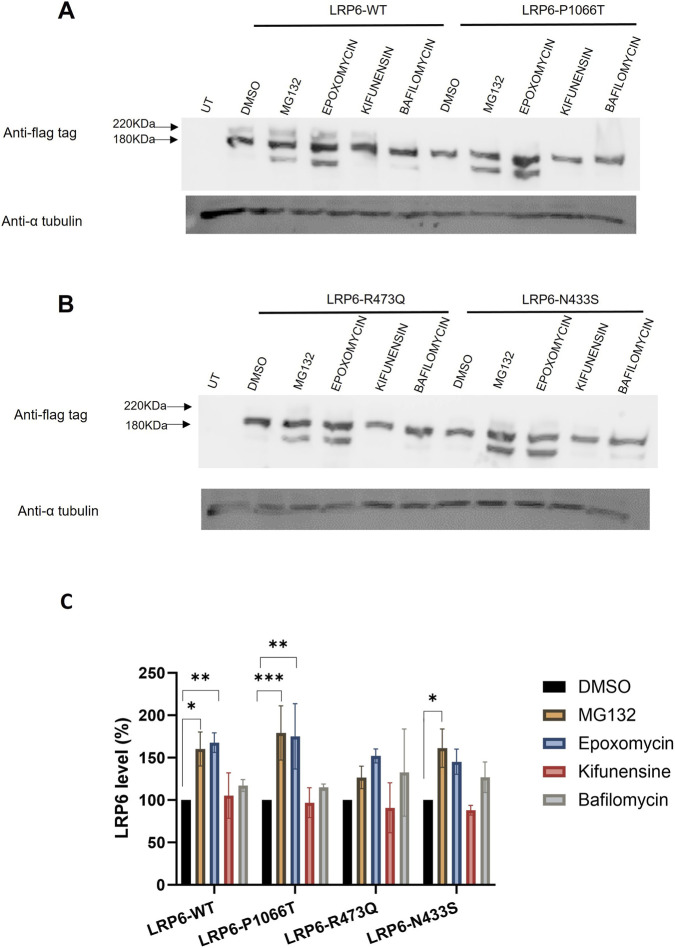
*Effect of different inhibitors on the degradation rates of LRP6 WT and mutants.* **(A,B)** HEK293T cells transiently expressing the LRP6 WT/mutants were treated with bafilomycin (200 nM) (lysosomal inhibitor), kifunensine (50 nM) (ERAD inhibitors), MG132 (10 μM), and epoxomycin (100 nM) (proteasomal inhibitors). Total cell lysate was analyzed by immunoblotting against antibodies for Flag tag and alpha tubulin. **(C)** Bar graphs representing mean densities of LRP6 WT and mutants normalized with alpha tubulin. The LRP6 level was expressed in (%) relative to DMSO-treated control. Error bars represent SEM from three different Experiments. Statistical significance for each treatment relative to DMSO was assessed using two-way ANOVA; (*) p ≤ 0.05; (**); p ≤ 0.01; (***) p ≤ 0.001.

Treatment with MG132 showed a significant increase in the accumulation of WT LRP6 (1.6-fold), whereas the mutant P1066T showed 1.8-fold accumulation compared to the DMSO-treated cells. The accumulation was correspondingly lower in R473Q and N433S, at 1.3- and 1.6-fold, respectively (Figure 9). On the other side, treatment with epoxomicin, an epoxyketone-containing natural product, and a potent, selective, and irreversible proteasome inhibitor showed a significant increase in the accumulation of WT LRP6 by 1.7-fold and P1066T by 1.8-fold, whereas other mutants R473Q and N433S showed 1.5-fold accumulation (Supplementary Image 4). The additional lower-molecular-weight bands observed following MG132 and epoxomicin treatment likely represent degradation intermediates or partially processed proteolytic fragments; however, their exact identity remains unclear.

Furthermore, treatment with bafilomycin, a lysosomal inhibitor that is known as a strong inhibitor of the vacuolar-type H (+)-ATPase *in vitro*, resulted in a non-significant accumulation level for LPR6 WT and all the mutants. Kifunensine, a potent inhibitor of mannosidase I enzyme, inhibits both human endoplasmic reticulum α-1,2-mannosidase I and members of the Golgi subfamily of the class I mannosidases. The treatment with kifunensine has not affected the steady-state levels of the LRP6 WT or the mutant variants.

Together, our results indicate that the ER-retained LRP6 mutants P1066T, R473Q, and N433S are likely to be subject to degradation via the proteasomal pathway, but further experiments are required to further elucidate the degradation pathway.

## 4 Discussion

CVDs are comprehended as a main leading cause of death worldwide that encompass a wide range of progressive disorders and associated risk factors (Kathiresan and Srivastava, 2012). A leading risk factor that is gaining increased attention in recent years is the genetic inheritance associated with CVD events (Kathiresan and Srivastava, 2012; Ylä-Herttuala and Baker, 2017). Genetic variations in the LDLR protein family members are linked to the development of atherosclerosis, MetS, and CVDs (Go and Mani, 2012; Mineo, 2020). Mutations in the LRP6, a member of this protein family, were reported in patients presented with multiple MetS features and early-onset CADs (Xu et al., 2014; Singh et al., 2013; Mani et al., 2007; Guo et al., 2016). In this study, we aimed to delineate the underlying effects of 10 LRP6 genetic variants on the cellular behavior of LRP6.

The LRP6 extracellular structure includes four EGF homology regions and four β-propeller domains, which are configured into four pairs, each consisting of two functional units, P1E1P2E2 and P3E3P4E4 (Bourhis et al., 2010). The P1E1P2E2 unit provides a binding site for the Wnt9b ligand, while the P3E3P4E4 unit binds to the Wnt3a ligand (Bourhis et al., 2010). Both Wnt9b and Wnt3a play an essential role in facial development (Imai and Lehmann, 1975; Jin et al., 2020). Additionally, Wnt3a is involved in the regulation of endothelial cell proliferation and migration (Samarzija et al., 2009). P3E3P4E4 also interacts with the Wnt signaling antagonist, DKK1 (Bourhis et al., 2010; Chen et al., 2011), which causes the internalization of LRP6 (Yamamoto et al., 2008). Another component of the LRP6 extracellular domain are the three LDLR Type A repeats, which mediate the transduction of Wnt signals and the endocytosis of LRP6 (Vijayakumar et al., 2017). The studied LRP6 genetic variants were all located on three extracellular regions of the protein (Figure 1). A summary of the studied mutation sites in both DNA and protein sequences is provided in Table 3.

**TABLE 3:** List of the mutation sites in the gene and protein sequences.

Mutation site in the gene sequence		Mutation site in the protein sequence	
c.246A>T	Exon 2	p. Lys82Asn	First β-propeller domain
c.1079G>A	Exon 6	p. Arg360His	Second β-propeller domain
c.1252T>C	Exon 6	p. Tyr418His	Second β-propeller domain
c.1298A>G	Exon 6	p. Asn433Ser	Second β-propeller domain
c.1418G>A	Exon 7	p. Arg473Gln	Second β-propeller domain
c.1463C>A	Exon 7	p. Ser488Tyr	Second β-propeller domain
c.1831C>T	Exon 9	p. Arg611Cys	Second EGF domain
c.3196C>A	Exon 14	p. Pro1066Thr	Fourth β-propeller domain
c.3617C>A	Exon 17	p. Pro1206His	Fourth β-propeller domain
c.3790A>G	Exon 18	p. Ile1264Val	First LDLR Type A repeats

Genetic variations in some members of the LDLR protein family were elaborated to be causing deficient cellular trafficking of the protein and activation of the ER stress response. Approximately 50% of LDLR variations causing familial hypercholesterolemia acclaim deficient cellular trafficking and activate the ER stress sensors IRE1 and PERK (Sørensen et al., 2006), which initiate UPR and proteasomal degradation of the protein (Oommen et al., 2020; Li et al., 2004). Similar events are observed in VLDLR variants that cause disequilibrium syndrome, which are retained in the ER and interact with SEL1L, an ERAD component, leading to proteasomal degradation of the mutant protein (Kizhakkedath et al., 2018; Kizhakkedath et al., 2014). Moreover, studies on genetic variants of LRP6 associated with autosomal dominant oligodontia (Massink et al., 2015) and autosomal dominant tooth agenesis (xia et al., 2021) demonstrated that these variants also cause loss of function due to impaired maturation and cellular localization of the protein. The accumulation of misfolded LRP6 variants in the ER can disrupt normal LRP6 protein folding homeostasis and lead to ER stress. When misfolded proteins accumulate beyond the ER’s folding capacity, they trigger the unfolded protein response (Varghese et al., 2023). This research provides evidence that some CVD-associated LRP6 variants also cause ER retention of the protein that trigger an ER stress response.

The variant showing ER retention (Y418H, R473Q, and P1066T) had a significantly lower maturation rate than the wild-type protein. This finding provides a partial explanation for the association of Y418H and R473Q mutations with impaired Wnt3a signaling (Singh et al., 2013; Guo et al., 2016) and the association of P1066T with impaired Wnt1 signaling (Xu et al., 2014), thereby causing deficient endothelial proliferation and migration (Xu et al., 2014; Singh et al., 2013; Guo et al., 2016). The protein dysfunction caused by the P1066T variant was demonstrated to result from the full retention of the protein (Figure 4), due to the formation of new bonds that caused a structural difference between wild-type LRP6 and P1066T (Figure 7). The R611C variant was the first to be identified as a CVD-associated mutation in LRP6 (Mani et al., 2007). Since then, it has been thoroughly studied and used as a model to provide a better understanding of the LRP6 functions and was demonstrated to induce the dysfunction of Wnt3a signaling (Go et al., 2014), LDL clearance (Liu et al., 2008), and PDGFR-β signaling (Keramati et al., 2011). The *in silico* studies suggested that the impaired function of R611C is correlated to the loss of an important salt bridge between the mutated arginine and Asp477 (Figure 6).

Further research could unravel the ER stress and ERAD components that interact with these ER-retained mutants. Elucidating these cellular events could lead to the development of therapeutic drugs that could rescue the ER-retained protein from degradation and restore the function of the protein. Within the recent past, there has been a growing interest in the possibility of developing drugs that modulate the rescue of misfolded proteins from ERAD. For example, pharmacological chaperones, which are small molecules that bind to the proteins in the ER and stabilize them, are being developed to effectively protect misfolded proteins from the ER quality control mechanism undergoing proteasomal degradation (Oommen et al., 2020; Ringe and Petsko, 2009). Other drugs, called proteostasis regulators, increase the function and availability of ER chaperones, thereby increasing the protein folding efficiency and rescuing the misfolded proteins from causing ER stress and activating UPR (Wang et al., 2011; Gámez et al., 2018).

Upon exploring the effect of these variants on the cellular localization and glycosylation profile of LRP6, four other variants (K82N, R360H, S488Y, and I1264V) confirmed to exhibit similar localization patterns to the wild type and showed no significant change in the SDS-PAGE and glycosylation patterns of the protein. Functional analysis of K82N, S488Y, and I1264V detected a significant decrease in the binding ability of the mutant proteins to Wnt1, which resulted in reduced proliferation and migration of endothelial cells (Xu et al., 2014). Although these mutations did not affect the folding and export of LRP6 to the plasma membrane, they impaired Wnt signaling and caused endothelial dysfunction, both of which are strongly associated with CVD pathogenesis (Gay and Towler, 2017; Hadi et al., 2005). However, in this study, non-disease-relevant cell lines such as Hela and HEK293T were used; therefore, the findings may not fully reflect physiological conditions, and their relevance to disease contexts should be investigated thoroughly.

Potentially, the function of these LRP6 variants that do not cause ER retention could be restored by treatment with Wnt3a, based on the finding that the effect of partial retention and impaired signaling of LRP6 caused by the R611C variant was evidently reversed by pharmacological intervention with Wnt3a. Treatment of mice expressing R611C LRP6 variants with Wnt3a led to normalization of LDL levels by reducing IGF1–Sp1–mTOR–SREBP1/2 signaling (Go et al., 2014). Moreover, Wnt3a treatment increased the signaling of TCF7L2 and regulates VSMC proliferation and differentiation in these mice (Srivastava et al., 2015). Therefore, with the aim of identifying therapeutic candidates, it is worthy to explore the possibility of Wnt3a as a therapeutic agent to restore the function of LRP6 variants that are not ER-retained but display impaired Wnt signaling.

## 5 Conclusion

Relying on genetic strategies for the diagnosis and therapy of CVD will only become possible in the future if the research work continues to uncover the underlying pathophysiology of altered gene expression and genetic mutations in the proteins, regulating the lipid uptake, cellular functions of endothelial cells, and other cardiovascular-protective functions. Genotyping in CVD risk prediction can be a powerful tool for family screening and diagnosis. Ten missense variants of LRP6 linked to early development of CVDs and comorbidities characteristic of MetS features were generated to study the consequence of its altered protein expression and localization. The results observed from immunofluorescence imaging of transfected HeLa cells indicated that some variants were abnormally retained in the ER and thus exhibited reduced localization on the plasma membrane. Western blotting detected two bands that correspond to the lighter, immature protein and the fully glycosylated, mature protein in HEK293T cell lysates overexpessing WT LRP6. The pattern of separation of the LRP6 protein in cells expressing the variants K82N, R360H, N433S, S488Y, R611C, P1206H, and I1264V was similar to that of wild type. On the other hand, the separation of protein bands for Y418H, R473Q, and P1066T variants was disparate in terms of the proportion of the higher-molecular- (mature) and lower-molecular (immature)-weight bands and alteration in the glycosylation profiles. The decay profile observed suggests that LRP6 stability may be modulated by various cellular factors, possibly including post-translational modifications or interactions with E3 ligases. To evaluate the possibility of rescuing the LRP6 variants from ERAD degradation, gene silencing strategies targeting the associated ERAD components may provide interesting leads that re-instate their ER–Golgi–plasma membrane transport and partially restore LRP6 function.

## Data Availability

The raw data supporting the conclusions of this article will be made available by the authors, without undue reservation.
