# Supplementary figures and images for: Evaluation of the cellular impact of missense variants in low-density lipoprotein receptor-related protein 6 (LRP6) associated with cardiovascular diseases in HeLa and HEK293T cell lines
Source: Front Cell Dev Biol. 2026 Jul 9;14:1828772. doi: 10.3389/fcell.2026.1828772 (PMC13391902; doi:10.3389/fcell.2026.1828772)

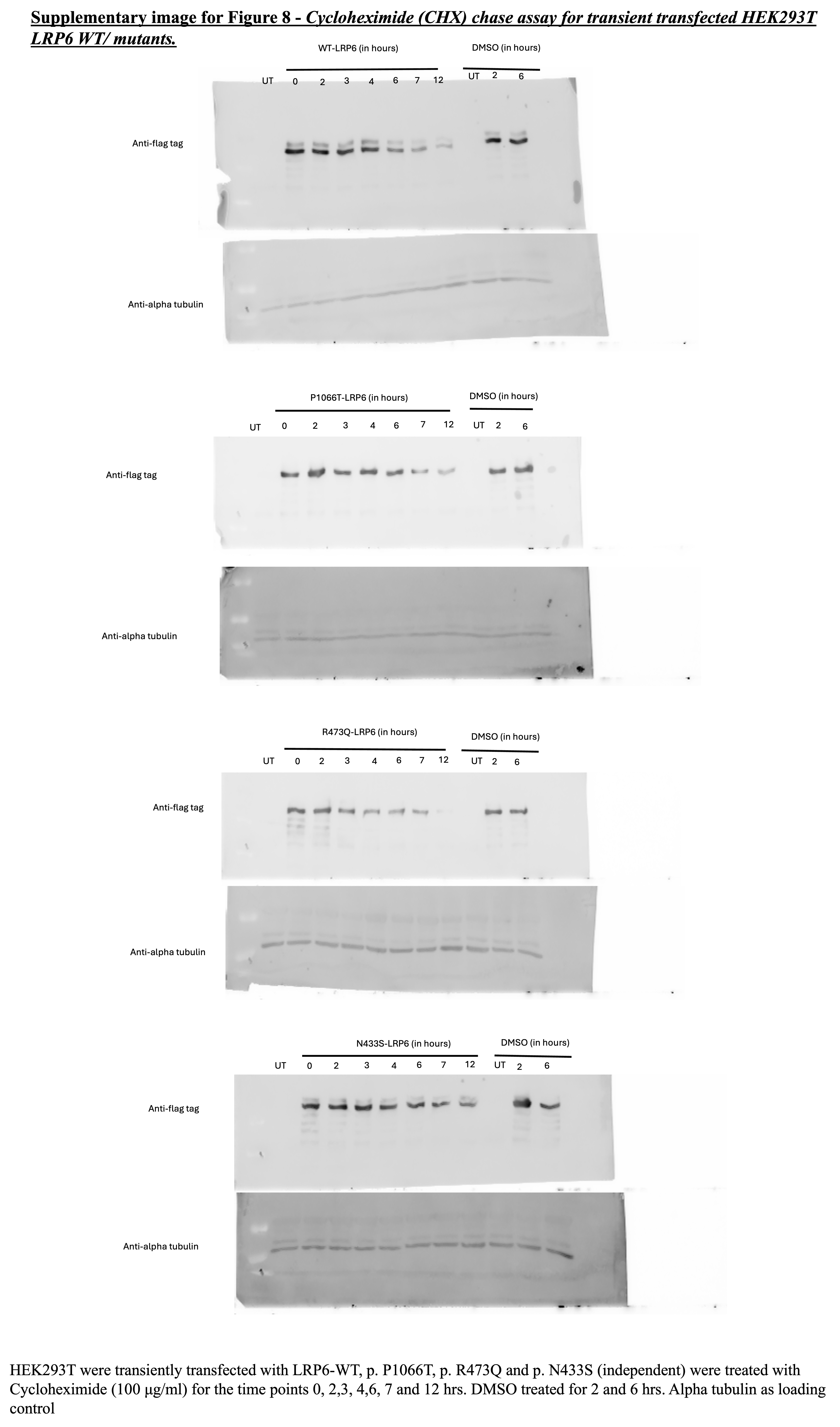

Supplement: Supplementary file 1 [file Image3.jpeg]

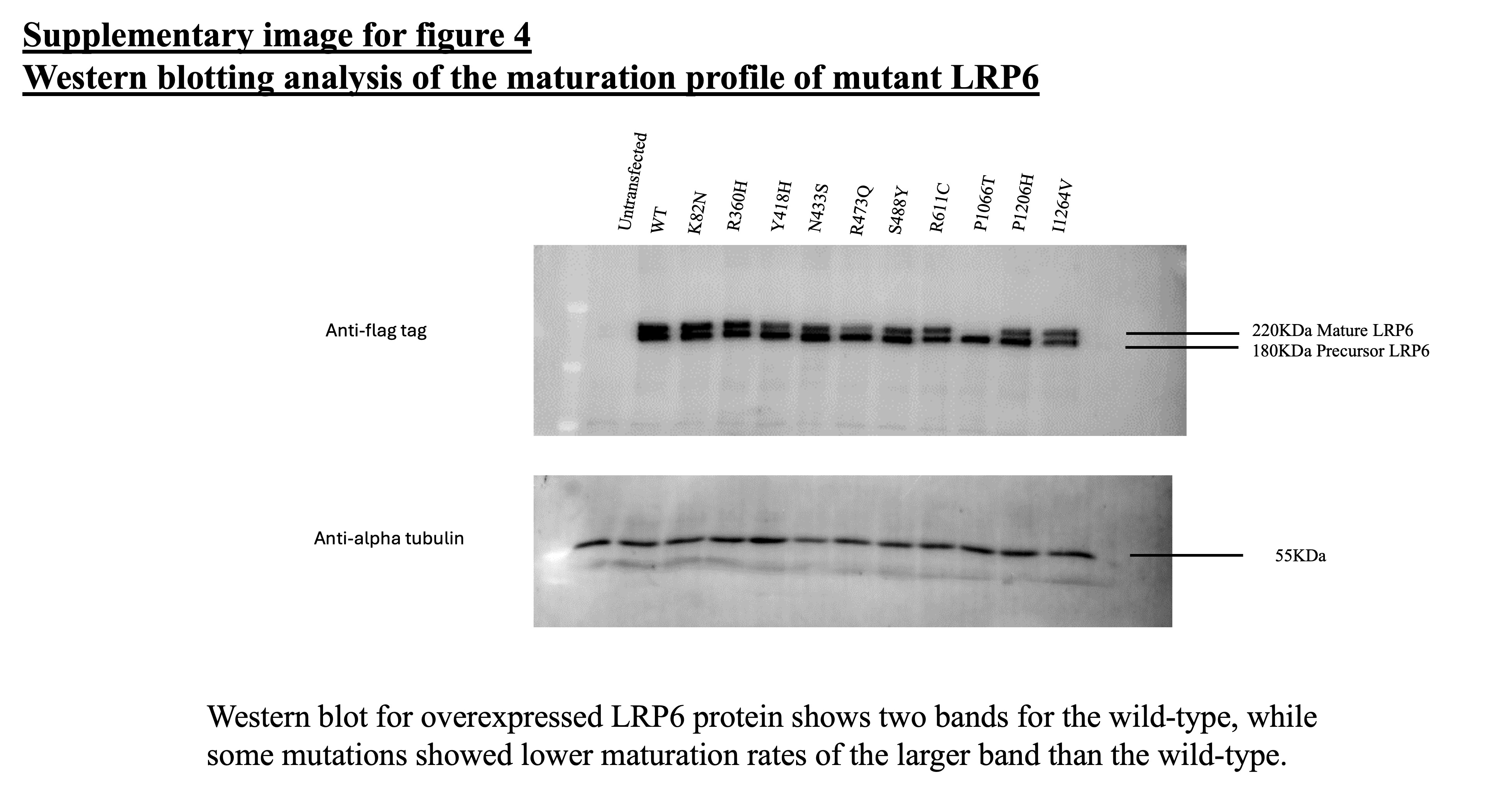

Supplement: Supplementary file 3 [file Image1.jpeg]

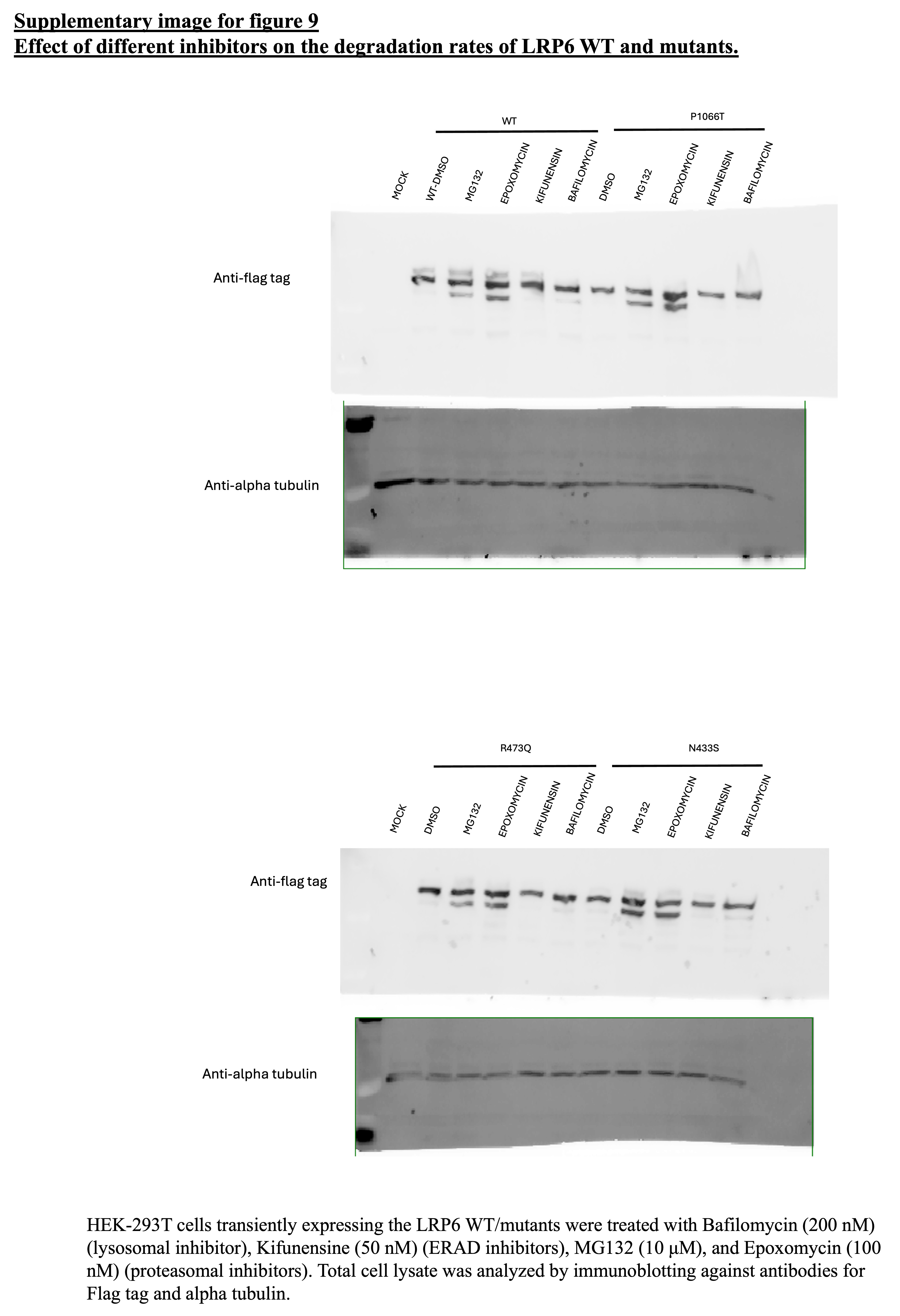

Supplement: Supplementary file 4 [file Image4.jpeg]

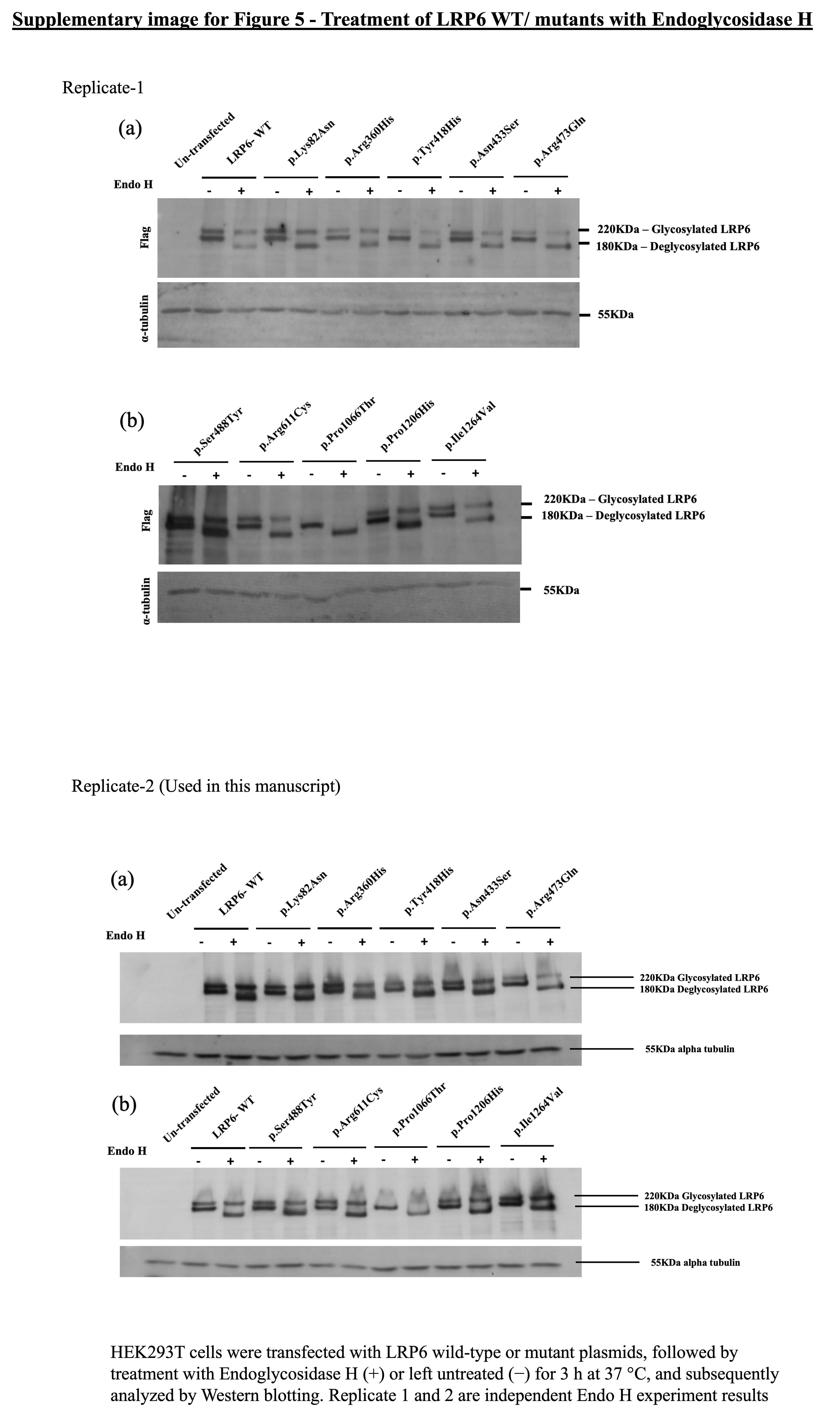

Supplement: Supplementary file 5 [file Image2.jpeg]
