# Supplementary material for: Evaluation of the cellular impact of missense variants in low-density lipoprotein receptor-related protein 6 (LRP6) associated with cardiovascular diseases in HeLa and HEK293T cell lines
Source: Front Cell Dev Biol. 2026 Jul 9;14:1828772. doi: 10.3389/fcell.2026.1828772 (PMC13391902; doi:10.3389/fcell.2026.1828772)
Supplement: Supplementary file 2 [file Table1.docx]

**Supplementary Table 1- LRP6 variants and the mutagenic primers designed using Primer X.**

| **LRP6 Mutation** | **Mutagenic Primers** |
| --- | --- |
| K82N | F: 5' GAACAGAATTTAACAA**T**ACTGAGAGTGTGCAG 3' |
|  | R: 5' CTGCACACTCTCAGT**A**TTGTTAAATTCTGTTC 3' |
| R360H | F: 5' GCAGTTAGAAGACATCC**A**TCATGCCATTGCCATAG 3' |
|  | R: 5' CTATGGCAATGGCATGA**T**GGATGTCTTCTAACTGC 3' |
| Y418H | F: 5' GTTGCACGAAATCTT**C**ATTGGACAGACACTG 3' |
|  | R: 5' CAGTGTCTGTCCAAT**G**AAGATTTCGTGCAAC 3' |
| N433S | F: 5' GTGACAAGGCTCA**G**TGGGACCATGAGG 3' |
|  | R: 5' CCTCATGGTCCCA**C**TGAGCCTTGTCAC 3' |
| R473Q | F: 5' GAAATTCCGAAAATTGAGC**A**AGCAGCTCTGGATGGTTC 3' |
|  | R: 5' GAACCATCCAGAGCTGCT**T**GCTCAATTTTCGGAATTTC 3' |
| S488Y | F: 5' GTATTGGTTAACACTT**A**TCTTGGTTGGCCAAATG 3' |
|  | R: 5' CATTTGGCCAACCAAGA**T**AAGTGTTAACCAATAC 3' |
| R611C | F: 5' CTATAGACCTCAGGGCCTT**T**GCTGTGCTTGCCCTATTGG 3' |
|  | R: 5'CCAATAGGGCAAGCACAGC**A**AAGGCCCTGAGGTCTATAG 3' |
| P1066T | F: 5' GAGCCGTTGTGGTAAAC**A**CAGAGAAAGGGTATATG 3' |
|  | R: 5' CATATACCCTTTCTCTG**T**GTTTACCACAACGGCTC 3' |
| P1206H | F: 5' GAATACAGACAGCACC**A**TTGTGCTCAGGATAATG 3' |
|  | R: 5' CATTATCCTGAGCACAA**T**GGTGCTGTCTGTATTC 3' |
| I1264V | F: 5' GGGAAATTGACTGT**G**TCCCTGTGGCTTGG 3' |
|  | R: 5' CCAAGCCACAGGGA**C**ACAGTCAATTTCCC 3' |

F: forward primer, R: reverse primer. The mutagenic nucleotides are indicated by red color bold font.

**Supplementary Table 2-List of LRP6 sequencing primers.**

| **LRP6 Sequencing primers** | **Primer sequence** |
| --- | --- |
| Sequencing Primer 1 | F: 5' CTTGCGATTGGTTGATGCTA 3' |
|  | R: 5' GCACTTCTCCCCAGTCTGTC 3' |
| Sequencing Primer 2 | F: 5' GGAAAAACCTGCAAAGATGG 3' |
|  | R: 5' CCTCTGCCAGTCAGTCCAGT 3' |
| Sequencing Primer 3 | F: 5' ACTGGACTGACTGGCAGAGG 3' |
|  | R: 5' TCGGTTGTCTGTCACATCAAA 3' |
| Sequencing Primer 4 | F: 5' CCCTATGACCTCAGCATTGA 3' |
|  | R: 5' CAGCCTGCTATCAAGGGCTA 3' |
| Sequencing Primer 5 | F: 5' TGGATTGATAAACAGCAGCAA 3' |
|  | R: 5' TGATTTGTCCTGGCAGTTTG 3' |

F: forward primer, R: reverse primer.
